# Migrationshintergrund und Einsamkeit im mittleren und hohen Alter in Deutschland

**DOI:** 10.1007/s00103-024-03923-4

**Published:** 2024-08-07

**Authors:** André Hajek, Hans-Helmut König

**Affiliations:** Institut für Gesundheitsökonomie und Versorgungsforschung, Universitätsklinikum Hamburg-Eppendorf, Hamburg Center for Health Economics, Martinistraße 52, 20246 Hamburg, Deutschland

**Keywords:** Einwanderer, Einsamkeit, Soziale Isolation, Soziale Ausgrenzung, Soziale Abgeschiedenheit, Ältere, Mittleres Alter, Immigrant, Loneliness, Social isolation, Social exclusion, Social disconnectedness, Older adults, Middle-aged

## Abstract

**Hintergrund:**

Bisher gibt es nur wenige Erkenntnisse über den Zusammenhang zwischen Migrationshintergrund und Einsamkeit im mittleren und hohen Alter in Deutschland. Ziel war daher, eine Assoziation zwischen Migrationshintergrund und Einsamkeit in dieser Gruppe darzustellen.

**Methoden:**

Die Daten stammen aus dem Deutschen Alterssurvey (Welle 7, November 2020 bis März 2021), einer repräsentativen Stichprobe von zuhause lebenden Personen mittleren und höheren Alters. Die Stichprobe umfasste 4145 Individuen. Das mittlere Alter betrug 63,8 Jahre, 93,2 % der Befragten hatten keinen Migrationshintergrund, wohingegen ungefähr 5,9 % der Befragten einen Migrationshintergrund mit eigener Migrationserfahrung und 0,9 % einen Migrationshintergrund, aber ohne eigene Migrationserfahrung hatten. Zur Quantifizierung der Einsamkeit wurde das etablierte Instrument von De Jong Gierveld verwendet.

**Ergebnisse:**

Multiple lineare Regressionen zeigten, dass Personen mit Migrationshintergrund und eigener Migrationserfahrung im Vergleich zu Personen ohne Migrationshintergrund eine signifikant höhere Einsamkeit aufweisen (β = 0,15, 95 % Konfidenzintervall (KI): 0,004–0,30, *p* < 0,05), wohingegen Personen mit Migrationshintergrund, aber ohne eigene Migrationserfahrung eine signifikant niedrigere Einsamkeit aufweisen (β = −0,27, 95 % KI: −0,52 bis −0,02, *p* < 0,05).

**Diskussion:**

Personen mit Migrationshintergrund und eigener Migrationserfahrung scheinen eine Risikogruppe für hohe Einsamkeit im mittleren und hohen Alter in Deutschland darzustellen. Insofern sollte diese Gruppe bei entsprechenden Maßnahmen besonders berücksichtigt werden. Vor dem Hintergrund der aktuellen (und potenzieller künftiger) Migrationsbewegungen sind diese Ergebnisse von großer Relevanz, da insbesondere diese Gruppen von Einsamkeit betroffen sein könnten.

**Zusatzmaterial online:**

Zusätzliche Informationen sind in der Online-Version dieses Artikels (10.1007/s00103-024-03923-4) enthalten.

## Abstract (en)

**Background:**

Currently, there is limited knowledge about the association between a migration background and loneliness among middle-aged and older individuals in Germany. The aim was therefore to examine the association between migration background and loneliness in this group.

**Methods:**

Data were taken from the German Ageing Survey (Wave 7, November 2020 to March 2021), a representative sample of middle-aged and older individuals. The sample comprised 4145 individuals, and the mean age was 63.8 years. Of the respondents, 93.2% had no migration background, approximately 5.9% had a migration background with personal migration experience, and 0.9% had a migration background but no personal migration experience. The De Jong Gierveld tool was used to quantify loneliness.

**Results:**

Multiple linear regressions showed that individuals with a migration background and their own migration experience have significantly higher levels of loneliness (β = 0.15, 95% confidence interval (CI): 0.004 to 0.30, *p* < 0.05) compared to individuals without a migration background, whereas individuals with a migration background without their own migration experience have significantly lower levels of loneliness (β = −0.27, 95% CI: −0.52 to −0.02 *p* < 0.05).

**Conclusions:**

Individuals with a migration background and their own migration experience appear to represent a risk group for high loneliness among middle-aged and older adults in Germany. In this respect, this group should be given special consideration in corresponding measures. Against the background of current (and potential future) migration movements, the results are of great importance as these groups in particular could be affected by loneliness.

## Einleitung

Einsamkeit stellt ein Gefühl der Diskrepanz zwischen gewünschten und den tatsächlichen sozialen Beziehungen dar [1]. Einsamkeit wird oftmals als das „neue Rauchen“ bezeichnet, da sie Morbidität und Mortalität prädiziert [2–4]. Auch hängt Einsamkeit negativ mit gesundheitsbezogener Lebensqualität [5] und der Schlafqualität [6] zusammen und ist zudem mit erheblichen Krankheitskosten assoziiert [7]. Ebenso kann Einsamkeit die Wahrscheinlichkeit der Inanspruchnahme präventiver Leistungen reduzieren [8].

Einsamkeit tritt im höheren Alter häufig auf [9]. Als Risikofaktoren im Alter werden u. a. genannt: funktionelle Beeinträchtigungen [10], sensorische Beeinträchtigungen [11] und insbesondere auch der Verlust des Partners bzw. der Partnerin ([12]; s. auch [13]).

International wurden Unterschiede bezüglich der Einsamkeit in Abhängigkeit des Migrationshintergrundes schon vermehrt untersucht [14–16] – häufig mit dem Ergebnis höherer Einsamkeit bei Personen mit Migrationshintergrund im Vergleich zu Personen ohne Migrationshintergrund. Hierzulande ist allerdings nur wenig bekannt zur Assoziation zwischen Migrationshintergrund und Einsamkeit im mittleren und höheren Alter. Im Einklang mit jüngeren empirischen Analysen [9, 17] vermuten wir dabei eine positive Assoziation zwischen Einsamkeit und einem Migrationshintergrund (insbesondere wenn eine Migrationserfahrung vorliegt). Solch eine positive Assoziation könnte sich insbesondere dadurch begründen, dass Freunde und Familie aus der Heimat bzw. die kulturellen Werte der Heimat vermisst werden (siehe auch [18]). Ebenso könnten Erfahrungen der Ausgrenzung, Rassismus und Diskriminierung von Relevanz sein [19]. Erwähnenswert in diesem Kontext ist auch Berrys Modell der Akkulturationsstrategien ([20]; d. h., wenn Individuen mit unterschiedlichen Kulturen sich begegnen). Dieses Modell unterscheidet 4 Strategien: Integration (Beibehaltung der Ursprungskultur mit einem entsprechenden Kontakt zur dominierenden Kultur), Segregation (Beibehaltung der Ursprungskultur und Ablehnung von Einflüssen der dominierenden Kultur), Assimilation (Aufgabe der eigenen Kultur und entsprechender Austausch mit der dominierenden Kultur) und Marginalisierung (Aufgabe der eigenen Kultur und zugleich keine Kontakte zur dominierenden Kultur). Im Einklang mit früherer Forschung [15] ist grundsätzlich zu vermuten, dass insbesondere marginalisierte Gruppen hohe Einsamkeitswerte aufweisen.

Aufgrund der begrenzten Studienlage ist das Ziel unserer Arbeit die Untersuchung der Assoziation zwischen einem Migrationshintergrund und Einsamkeit. Vor dem Hintergrund der aktuellen (und möglicher künftiger) Migrationsbewegungen ist dies von entsprechender Relevanz: In Deutschland haben nach Angaben des Statistischen Bundesamtes derzeit 23,8 Mio. Menschen (bei einer Gesamtpopulation von ca. 83,1 Mio. Menschen) einen Migrationshintergrund [21]. Im Jahr 2020 hatten beispielsweise mehr als 2,2 Mio. Menschen ab 65 Jahren (von insgesamt ca. 17,3 Mio. Menschen in dieser Altersgruppe) einen Migrationshintergrund [22]. Davon hatten ca. 1,9 Mio. Menschen eine eigene Migrationserfahrung und ca. 0,3 Mio. Menschen hatten keine eigene Migrationserfahrung.

## Methoden

### Stichprobe

Zum Deutschen Alterssurvey (DEAS) im Allgemeinen: In der DEAS-Studie werden zuhause lebende Personen ab 40 Jahren (d. h. zweite Lebenshälfte) interviewt und bekommen anschließend einen schriftlichen Fragebogen mit sensibleren Themen wie Einsamkeit. Die DEAS-Studie ist für diese Gruppe repräsentativ. Den Fragebogen konnten die Befragten selbst ausfüllen. In beiden Fällen (sowohl im Interview als auch im Fragebogen) ist die Sprache ausschließlich Deutsch. Die DEAS-Studie hat ein kohortensequenzielles Design (siehe [23]). Solch ein Design setzt sich aus großen repräsentativen Querschnittstichproben und entsprechenden Längsschnittstichproben (d. h. Wiederholungsbefragungen) zusammen. Die DEAS-Studie erhielt Mittel vom Bundesministerium für Familie, Senioren, Frauen und Jugend (BMFSFJ). Ein wesentliches Ziel der DEAS-Studie ist, die zweite Lebenshälfte im Allgemeinen besser zu verstehen (z. B. Wohlbefinden, Gesundheit, Beruf oder auch Diskriminierung im Alter [23]).

Um möglichst aktuelle Daten zu nutzen, wurden die Daten für diese Studie aus Welle 7 des DEAS verwendet. Die Feldarbeit für die Umfrage wurde vom Institut für angewandte Sozialwissenschaft (infas) durchgeführt und erstreckte sich von November 2020 bis März 2021. Die mittlere Dauer eines Interviews in Welle 7 betrug ungefähr 75 min. Die DEAS-Studie verwendete eine computergestützte persönliche Befragungsmethode (Computer Assisted Personal Interview: CAPI), die in dieser Welle wegen der COVID-19-Pandemie nur telefonisch durchgeführt wurde. Darüber hinaus erhielten die Befragten den genannten schriftlichen Fragebogen.

Aufgrund der pandemischen Lage wurde in Welle 7 auf eine neu gezogene Baseline-Stichprobe verzichtet. Daher bestand die Stichprobe für Welle 7 aus Personen, die aus den Baseline-Stichproben von 1996 bis 2014 erreichbar blieben und bereit waren, an der DEAS-Studie weiter teilzunehmen. Insofern waren in dieser Welle die Befragten mind. 46 Jahre alt. Die Rücklaufquote in Welle 7 betrug ungefähr 65 % (basierend auf den Telefoninterviews). Insgesamt füllten 4419 Befragte den schriftlichen Fragebogen aus. Insofern liegt für ca. 82 % der auswertbaren Telefoninterviews ein ausgefüllter schriftlicher Fragebogen vor. Aufgrund fehlender Werte bestand die analytische Stichprobe aus *n* = 4145 Befragten. Wesentlicher Grund für die Nichtteilnahme waren die generelle Weigerung, an der Umfrage teilzunehmen, und der damit verbundene Rückzug der Bereitschaft, Teil des Panels zu sein. Faktoren wie Größe der Gemeinde, Einkommenskategorie und Familienstand waren jedoch mehrheitlich nicht mit der Teilnahmewahrscheinlichkeit assoziiert [24]. Zusätzliche Details zur DEAS-Studie finden sich bei [23].

### Outcome: Einsamkeit

Als Zielgröße fungierte die Einsamkeit. Diese wurde mithilfe der 6‑Item-Variante der De-Jong-Gierveld-Einsamkeitsskala quantifiziert [25]. Insbesondere um möglichst einheitlich im DEAS-Fragebogen zu sein, wurden für jedes Item dieses Tools 4 Antwortmöglichkeiten vorgegeben (trifft genau zu; trifft eher zu; trifft eher nicht zu; trifft gar nicht zu). Drei der 6 Items wurden rekodiert bevor ein Mittelwert gebildet wurde. Die finale Skala reicht von 1 bis 4, wobei höhere Werte eine höhere Einsamkeit reflektieren (siehe [26]). Diese finale (kontinuierliche) Skala diente als Outcome in unserer vorliegenden Studie. In unserer Studie war Cronbachs Alpha 0,80 (McDonalds Omega war 0,81). Gute psychometrische Eigenschaften dieser Skala wurden auch in früheren Studien demonstriert [27].

### Migrationshintergrund

Der Migrationshintergrund basiert jeweils auf den Informationen aus dem ersten Interview. Die Definition orientiert sich am Konzept des Mikrozensus. Drei Gruppen werden dabei unterschieden: (1) Befragte ohne Migrationshintergrund, (2) Befragte mit Migrationshintergrund (und eigener Migrationserfahrung, d. h. Einwanderung nach Deutschland) sowie (3) Befragte mit Migrationshintergrund (ohne eigene Migrationserfahrung, d. h. in Deutschland geboren und aufgewachsen). In Kürze: Gruppe 2 sind Einwandererinnen und Einwanderer und Gruppe 3 sind Kinder von Einwandererinnen und Einwanderern (sie sind in Deutschland geboren und aufgewachsen, aber mindestens ein Elternteil hat Migrationserfahrung).

Für die Definition der Variable Migrationshintergrund wurden Angaben zum Geburtsort, zum Jahr der Zuwanderung, zum Besitz der deutschen oder einer ausländischen Staatsangehörigkeit und zur Erfahrung der Einbürgerung verwendet. Zuwanderungen vor 1950 gelten dabei nicht als Migrationshintergrund. Personen, die in den früheren deutschen Ostgebieten geboren wurden und nach 1949 zugewandert sind, zählen als Zuwandererinnen und Zuwanderer.

### Kovariaten

Im Einklang mit früheren Studien (z. B. [13, 28]) und theoretischen Überlegungen wurden die Kovariaten ausgewählt. Es wurden folgende soziodemografische Größen im Regressionsmodell berücksichtigt: Alter, Geschlecht (Mann; Frau), Familienstand (verheiratet, zusammenlebend; verheiratet, getrennt lebend; geschieden; verwitwet; Single), Erwerbsstatus (erwerbstätig; verrentet; sonstige nicht Erwerbstätige), Bildungsstand (International Standard Classification of Education[ISCED]-Klassifikation [29]: niedrig, mittel, hoch) sowie das Haushaltsnettoeinkommen in Euro.

Bezüglich des Lebensstils wurde im Regressionsmodell berücksichtigt: Häufigkeit sportlicher Aktivitäten, Alkoholkonsum (in beiden Fällen: täglich; mehrmals die Woche; einmal die Woche; ein- bis dreimal im Monat; seltener; nie) sowie der Rauchstatus (ja, täglich; ja, gelegentlich; nein, nicht mehr; nein, noch nie).

Hinsichtlich der Gesundheitsfaktoren wurde im Regressionsmodell berücksichtigt: subjektive Gesundheit (von 1 = sehr gut bis 5 = sehr schlecht), depressive Symptome (15-Item-Version der Center for Epidemiologic Studies Depression Scale [30]; 0 bis 45, höhere Werte bedeuten mehr depressive Symptome) und die Anzahl chronischer Krankheiten (von 0 bis 11 chronische Krankheiten, z. B. Diabetes mellitus oder Krebs).

### Statistische Analyse

Zunächst wurde die analytische Stichprobe dargestellt. Nach der Deskription der Stichprobe folgten unadjustierte und adjustierte lineare Regressionen, um die Assoziation zwischen Migrationshintergrund und Einsamkeit zu beleuchten. In den Hauptanalysen wurde ein fallweiser Ausschluss zur Adressierung fehlender Werte gewählt. In Sensitivätsanalysen wurde ein Full-Information-Maximum-Likelihood-(FIML-)Ansatz [31] gewählt. Bezogen auf die Teilnahme am schriftlichen Fragebogen hatten 12 der 14 Variablen meist deutlich weniger als jeweils 1 % fehlende Werte (z. B. Einsamkeit mit 0,5 % fehlenden Werten). Die Variable zur Messung der Anzahl chronischer Krankheiten hatte 1,3 % fehlende Werte und die Variable zur Messung des Haushaltsnettoeinkommens hatte 3,5 % fehlende Werte. Knapp 94 % der Befragten hatten bei allen Variablen Angaben gemacht.

Zur Adressierung des komplexen Stichprobendesigns wurden Gewichte (d. h. poststratifiziertes integriertes Querschnittgewicht, basierend auf dem schriftlichen Fragebogen) eingesetzt [24]. Details finden sich in Kapitel 7 des entsprechenden Methodenberichts [24] und hier [32]. Zu erwähnen ist auch: Aufgrund des demografischen Wandels in Deutschland werden nun auch Menschen im Alter von 91 bis 100 Jahren in ausreichender Zahl in den offiziellen Statistiken erfasst. Insofern konnten in Welle 7 die Gewichte auch die Bevölkerung bis inklusive 100 Jahre abbilden.

Robuste Standardfehler wurden in den Regressionsanalysen berechnet. Zur Berechnung von McDonalds Omega wurde „omegacoef“ [33] eingesetzt.

Als Grenze zur statistischen Signifikanz wurde α = 0,05 gewählt. Für die Datenanalyse wurde Stata 18.0 (Stata Corp., College Station, Texas) genutzt.

## Ergebnisse

### Stichprobenbeschreibung

Das mittlere Alter der Stichprobe war 63,8 Jahre (Standardabweichung (SD): 11,3 Jahre, 46 bis 98 Jahre). Insgesamt waren 51,4 % der Befragten weiblich. Der mittlere Einsamkeitswert betrug 1,8 (SD: 0,5). Es hatten ungefähr 93,2 % der Befragten keinen Migrationshintergrund, wohingegen ungefähr 5,9 % der Befragten einen Migrationshintergrund mit eigener Migrationserfahrung und 0,9 % der Befragten einen Migrationshintergrund, aber ohne eigene Migrationserfahrung hatten. Weitere Details finden sich in Tab. 1. Eine stratifizierte Darstellung nach Migrationshintergrund findet sich in Tabelle A1 im Onlinezusatzmaterial.

**Tab. 1:** Beschreibung der analytischen Stichprobe (*n* = 4145, gewichtet)

Variablen	Mittelwert (SD)/*n* (%)
Einsamkeit (De-Jong-Gierveld-Skala, 1 bis 4, je höher, desto größer die Einsamkeit)	1,8 (0,5)
Migrationshintergrund	
– Ohne Migrationshintergrund	3863 (93,2 %)
– Mit Migrationshintergrund und eigener Migrationserfahrung	246 (5,9 %)
– Mit Migrationshintergrund, aber ohne eigene Migrationserfahrung	36 (0,9 %)
Alter (in Jahren)	64,1 (11,5)
Geschlecht	
– Mann	2006 (48,4 %)
– Frau	2139 (51,6 %)
Familienstand	
– Verheiratet, zusammenlebend	2786 (67,2 %)
– Verheiratet, getrennt lebend	64 (1,5 %)
– Geschieden	446 (10,8 %)
– Verwitwet	538 (13,0 %)
– Ledig	310 (7,5 %)
Bildungsniveau nach ISCED-97	
– Niedrig (ISCED-97: 0–2)	427 (10,3 %)
– Mittel (ISCED-97: 3–4)	2130 (51,4 %)
– Hoch (ISCED-97: 5–6)	1588 (38,3 %)
Erwerbsstatus	
– Aktiv erwerbstätig	1994 (48,1 %)
– Im Ruhestand	1936 (46,7 %)
– Sonstige nicht Erwerbstätige	215 (5,2 %)
Haushaltsnettoeinkommen in Euro	3647,9 (3852,4)
Sport machen	
– Täglich	403 (9,7 %)
– Mehrmals pro Woche	1192 (28,7 %)
– Einmal pro Woche	599 (14,4 %)
– Ein- bis 3‑mal im Monat	280 (6,8 %)
– Seltener	503 (12,1 %)
– Nie	1169 (28,2 %)
Derzeitiges Rauchverhalten	
– Ja, täglich	540 (13,0 %)
– Ja, gelegentlich	195 (4,7 %)
– Nein, nicht mehr	1545 (37,3 %)
– Habe noch nie geraucht	1865 (45,0 %)
Häufigkeit von alkoholischen Getränken	
– Täglich	388 (9,4 %)
– Mehrmals pro Woche	1118 (27,0 %)
– Einmal pro Woche	612 (14,8 %)
– Ein- bis 3‑mal im Monat	515 (12,4 %)
– Seltener	1058 (25,5 %)
– Nie	453 (11,0 %)
Subjektiver Gesundheitszustand (von 1 = sehr gut bis 5 = sehr schlecht)	2,4 (0,8)
Anzahl körperlicher Erkrankungen	2,5 (2,0)
Depressive Symptome (CES‑D; 0 bis 45: je höher der Wert, desto mehr depressive Symptome)	6,2 (5,8)

Stratifiziert nach dem Migrationshintergrund zeigten sich folgende mittleren Einsamkeitswerte: 1,8 (SD: 0,5) für Befragte ohne Migrationshintergrund, 2,0 (SD: 0,4) für Befragte mit Migrationshintergrund und eigener Migrationserfahrung sowie 1,4 (SD: 0,5) für Befragte mit Migrationshintergrund, aber ohne eigene Migrationserfahrung.

### Regressionsanalysen

Zunächst wurden unadjustierte lineare Regressionsanalysen durchgeführt (Tab. 2; 2. Spalte: mit fallweisem Ausschluss, um fehlende Werte zu adressieren; 3. Spalte: mit FIML, um fehlende Werte zu adressieren). Im Vergleich zu Personen ohne Migrationshintergrund weisen Personen mit Migrationshintergrund und eigener Migrationserfahrung eine signifikant höhere Einsamkeit auf (β = 0,14, *p* < 0,05). Hingegen weisen Personen mit Migrationshintergrund, aber ohne eigene Migrationserfahrung eine signifikant niedrigere Einsamkeit im Vergleich zu Personen ohne Migrationshintergrund auf (β = −0,38, *p* < 0,01). Die Sensitivitätsanalysen (FIML zur Adressierung fehlender Werte) zeigen identische Ergebnisse.

**Tab. 2:** Migrationshintergrund und Einsamkeit. Ergebnisse basierend auf Welle 7 der DEAS-Studie (unadjustierte lineare Regressionen)

	Einsamkeit (Umgang mit fehlenden Werten: fallweiser Ausschluss)	Einsamkeit (Umgang mit fehlenden Werten: FIML)
Migrationshintergrund: Migrationshintergrund und persönliche Migrationserfahrung (Referenzkategorie: kein Migrationshintergrund)	0,14* (0,02–0,26)	0,14* (0,02–0,26)
Migrationshintergrund und ohne persönliche Migrationserfahrung	−0,38** (−0,60 bis −0,15)	−0,38** (−0,60 bis −0,15)
R^2^	0,01	0,01
Zahl der Beobachtungen	4391	4398

Unstandardisierte Betakoeffizienten werden dargestellt, 95 %-Konfidenzintervalle in Klammern, *** *p* < 0,001, ** *p* < 0,01, * *p* < 0,05, + *p* < 0,10; Gewichtung wurde vorgenommen

Adjustierte Regressionsanalysen sind in Tab. 3 zu finden. Diese sind ähnlich im Vergleich zu den unadjustierten Befunden aus Tab. 2. Im Vergleich zu Personen ohne Migrationshintergrund weisen Personen mit Migrationshintergrund und eigener Migrationserfahrung eine signifikant höhere Einsamkeit auf (β = 0,15, *p* < 0,05), wohingegen Personen mit Migrationshintergrund, aber ohne eigene Migrationserfahrung eine signifikant niedrigere Einsamkeit aufweisen (β = −0,27, *p* < 0,05). Die Kovariaten sind in Tabelle A2 im Onlinezusatzmaterial dargestellt.

**Tab. 3:** Migrationshintergrund und Einsamkeit. Ergebnisse basierend auf Welle 7 der DEAS-Studie (adjustierte lineare Regressionen)

	Einsamkeit (Umgang mit fehlenden Werten: fallweiser Ausschluss)	Einsamkeit (Umgang mit fehlenden Werten: FIML)
Migrationshintergrund: Migrationshintergrund und persönliche Migrationserfahrung (Referenzkategorie: kein Migrationshintergrund)	0,15* (0,004–0,30)	0,15* (0,01–0,30)
Migrationshintergrund und ohne persönliche Migrationserfahrung	−0,27* (−0,52 bis −0,02)	−0,27* (−0,51 bis −0,02)
Kovariaten	✔	✔
R^2^	0,16	0,16
Zahl der Beobachtungen	4145	4190

Unstandardisierte Betakoeffizienten werden dargestellt, 95 %-Konfidenzintervalle in Klammern, *** *p* < 0,001, ** *p* < 0,01, * *p* < 0,05, + *p* < 0,10; Gewichtung wurde vorgenommen; folgende Kovariaten wurden in das Regressionsmodell aufgenommen: Alter, Geschlecht, Familienstand, Bildung, Erwerbsstatus, Haushaltsnettoeinkommen, Häufigkeit sportlicher Aktivität, Alkoholkonsum, Rauchstatus, subjektive Gesundheit, depressive Symptome und Anzahl chronischer Erkrankungen

## Diskussion

Ziel dieser Arbeit war es, den Zusammenhang eines Migrationshintergrunds und der Einsamkeit im mittleren und hohen Alter in Deutschland empirisch zu untersuchen. Kernergebnisse waren wie folgt: Regressionen zeigten, dass Personen mit Migrationshintergrund und eigener Migrationserfahrung im Vergleich zu Personen ohne Migrationshintergrund eine signifikant *höhere* Einsamkeit aufweisen. Zugleich weisen Personen mit Migrationshintergrund, aber ohne eigene Migrationserfahrung im Vergleich zu Personen ohne Migrationshintergrund eine signifikant *niedrigere* Einsamkeit auf.

Der Unterschied in der Einsamkeit zwischen mittelalten und älteren Personen ohne Migrationshintergrund und mittelalten und älteren Personen mit Migrationshintergrund und eigener Migrationserfahrung ist im Einklang zu jüngeren Studien aus Deutschland [9, 17]. Potenzielle Erklärungsansätze könnten sich insbesondere zu Zeiten der Pandemie u. a. auf Reiserestriktionen und damit verbundene unterbliebene soziale Kontakte beziehen [34]. Ebenso könnten vermehrte depressive Symptome bei Personen mit Migrationshintergrund den Zusammenhang erklären [35]. Allerdings wurde in dieser Studie dafür adjustiert (in unserer Studie waren signifikante Unterschiede in depressiven Symptomen zwischen den 3 Gruppen bivariat auch nicht gegeben). Auch könnte die Qualität der sozialen Beziehungen eine bedeutende Rolle spielen. Die betroffene Gruppe könnten entsprechende Netzwerke, ihre ursprüngliche Kultur oder enge Freunde bzw. Verwandte aus ihrer eigentlichen Heimat vermissen [9, 36]. Ebenso könnten schlechte Erfahrungen in der Gesundheitsversorgung (z. B. Sprachbarrieren oder Erfahrungen der Ablehnung) Gefühle der Einsamkeit verstärken [37]. Auch generelle Probleme wie Diskriminierung und Rassismus seien hier zu nennen [19]. Vermutlich könnten auch Gefühle der Marginalisierung von großer Relevanz sein [15]. Zu betonen ist überdies, dass die Befragten mindestens seit 2014 im Panel waren (d. h. spätestens seit 2014 in Deutschland waren). Man könnte vermuten, dass die Einsamkeitswerte bei Personen, die weniger als 6–7 Jahre in Deutschland leben, höher sein könnten. Dazu bedarf es weiterer Forschung.

Dagegen könnten die niedrigeren Einsamkeitswerte bei Personen im mittleren und hohen Alter mit Migrationshintergrund, aber ohne eigene Migrationserfahrung (im Vergleich zu Personen ohne Migrationshintergrund) sich wie folgt erklären: Sie könnten darauf hindeuten, dass diese Personen sich gut in der deutschen Gesellschaft zurechtfinden und entsprechende soziale Kontakte in Deutschland aufweisen. Eine entsprechende Integration könnte also vorsichtig vermutet werden. Möglicherweise werden Kontakte zu Verwandten aus dem Ausland kaum vermisst. Eine spekulative Erklärung könnte auch sein, dass sich diese Personen mit ihren Eltern vergleichen, die möglicherweise höhere Einsamkeitswerte aufweisen. Dieser für sie positive Vergleich könnte niedrigere Einsamkeitswerte begünstigen. Allerdings bedarf es weiterer Forschung, um diese Aussagen im Detail zu prüfen. Es ist auch einschränkend darauf hinzuweisen, dass die Fallzahl der inkludierten Personen mit Migrationshintergrund, aber ohne eigene Migrationserfahrung in dieser Studie klein ist. Aufgrund der genannten geringen Fallzahl sind diese Ergebnisse mit entsprechender Vorsicht zu interpretieren.

Einige Stärken und Limitationen dieser Arbeit sollten Erwähnung finden. Als Datengrundlage fungierte die bevölkerungsbasierte DEAS-Studie. Zahlreiche Kovariaten wurden in das Regressionsmodell inkludiert. Weiterführend wurden auch Modelle mit einem FIML-Ansatz geschätzt. Einschränkend ist, dass Individuen mit Migrationshintergrund in dieser Studie unterrepräsentiert sind. Künftige Analysen (bspw. stratifiziert nach dem mittleren und hohen Lebensalter) wären in Zukunft wünschenswert. Dies lässt sich vermutlich auch darüber begründen, dass entsprechende Deutschkenntnisse wesentlich für die Teilnahme an der DEAS-Studie sind. Man könnte vermuten, dass die wahren Effekte (insbesondere bei den Einwanderern) noch unterschätzt werden. Auch muss betont werden, dass die Gruppe der Individuen mit Migrationshintergrund insgesamt recht heterogen ist [38]. Weitere Forschung ist notwendig, um Einsamkeitswerte für entsprechende Subgruppen (mit aussagekräftigen Fallzahlen) zu identifizieren. Hierzu kann unserer Ansicht nach auch das Modell von Berry [20] einen wertvollen Beitrag leisten. Auch die Lebenslaufepidemiologie könnte vermutlich einen wichtigen Beitrag zum besseren Verständnis von Einsamkeit bei Personen mit Migrationshintergrund leisten [39]. Es ist zu betonen, dass die Daten zu Pandemiezeiten erhoben wurden. Die Übertragbarkeit dieser Ergebnisse auf die postpandemische Ära bleibt abzuwarten.

Zusammenfassend scheinen Personen mit Migrationshintergrund und eigener Migrationserfahrung eine Risikogruppe für höhere Einsamkeit im mittleren und hohen Alter in Deutschland darzustellen. Vor dem Hintergrund der aktuellen (und potenzieller künftiger) Migrationsbewegungen sind diese Ergebnisse von großer Relevanz.

Entsprechende Ansätze sind notwendig, um Einsamkeit bei genannten Risikogruppen zu begegnen. Eine kürzlich erschienene Studie [40] hat beispielsweise gezeigt, dass physische Aktivitäten insbesondere bei älteren Personen mit Migrationshintergrund (und eigener Migrationserfahrung) in Deutschland zur Reduktion von Einsamkeit beitragen können. Es bedarf jedoch weiterer Forschung, um zusätzliche geeignete Maßnahmen für die entsprechenden Risikogruppen zu eruieren.

## Supplementary Information

Tabelle A1. Beschreibung der analytischen Stichprobe – stratifiziert nach Migrationshintergrund (n=4.145, gewichtet)
Tabelle A2. Migrationshintergrund und Einsamkeit. Ergebnisse basierend auf Welle 7 der DEAS-Studie (adjustierte lineare Regressionen; alle Kovariaten werden dargestellt)
