# Supplementary material for: Migration background and loneliness among middle-aged and older adults in Germany
Source: Bundesgesundheitsblatt Gesundheitsforschung Gesundheitsschutz. 2024 Aug 7;67(10):1137–43. [Article in German] doi: 10.1007/s00103-024-03923-4 (PMC11424693; doi:10.1007/s00103-024-03923-4)
Supplement: Supplementary file 1 — Tabelle A1. Beschreibung der analytischen Stichprobe – stratifiziert nach Migrationshintergrund (n=4.145, gewichtet) Tabelle A2. Migrationshintergrund und Einsamkeit. Ergebnisse basierend auf Welle 7 der DEAS-Studie (adjustierte lineare Regressionen; alle Kovariaten werden dargestellt) [file 103_2024_3923_MOESM1_ESM.docx]

**Migrationshintergrund und Einsamkeit im mittleren und hohen Alter in Deutschland**

André Hajek^1^, Hans-Helmut König^1^

^1^ Institut für Gesundheitsökonomie und Versorgungsforschung, Universitätsklinikum Hamburg-Eppendorf, Hamburg Center for Health Economics, Hamburg, Deutschland

**Tabelle A1**. Beschreibung der analytischen Stichprobe – stratifiziert nach Migrationshintergrund (n=4.145, gewichtet)

|  | Ohne Migrationshintergrund | Mit Migrationshintergrund und eigener Migrationserfahrung | Mit Migrationshintergrund, aber ohne eigene Migrationserfahrung |
| --- | --- | --- | --- |
| Variablen | Mittelwert (SD) / n (%) | Mittelwert (SD) / n (%) | Mittelwert (SD) / n (%) |
| Einsamkeit (De Jong Gierveld-Skala, 1 bis 4, je höher, desto größer die Einsamkeit) | 1,8 (0,5) | 2,0 (0,4) | 1,4 (0,5) |
| Alter (in Jahren) | 64,5 (11,6) | 59,4 (8,8) | 61,6 (9,2) |
| Geschlecht |  |  |  |
| Mann | 1.890 (47,6%) | 92 (61,3%) | 11 (39,4%) |
| Frau | 2.076 (52,4%) | 58 (38,7%) | 18 (60,6%) |
| Familienstand |  |  |  |
| Verheiratet, zusammenlebend | 2.634 (66,4%) | 119 (79,7%) | 20 (70,0%) |
| Verheiratet, getrennt lebend | 66 (1,7%) | 0 (0,0%) | 0 (0,0%) |
| Geschieden | 425 (10,7%) | 16 (10,9%) | 5 (16,0%) |
| Verwitwet | 526 (13,3%) | 13 (8,3%) | 4 (14,1%) |
| Ledig | 315 (8,0%) | 2 (1,1%) | 0 (0,0%) |
| Bildungsniveau nach ISCED |  |  |  |
| Niedrig (ISCED 0-2) | 387 (9,8%) | 31 (20,6%) | 0 (0,0%) |
| Mittel (ISCED 3-4) | 2.053 (51,8%) | 72 (48,5%) | 9 (32,3%) |
| Hoch (ISCED 5-6) | 1.527 (38,5%) | 46 (31,0%) | 20 (67,7%) |
| Erwerbsstatus |  |  |  |
| Aktiv erwerbstätig | 1.854 (46,7%) | 101 (67,3%) | 18 (62,1%) |
| Im Ruhestand | 1.903 (48,0%) | 42 (27,9%) | 11 (37,9%) |
| Sonstige nicht Erwerbstätige | 209 (5,3%) | 7 (4,8%) | 0 (0,0%) |
| Haushaltsnettoeinkommen in Euro | 3.592 (3.897,4) | 4.221 (2.991,3) | 5.758 (3.367,7) |
| Sport machen |  |  |  |
| Täglich | 370 (9,3%) | 16 (10,9%) | 12 (42,6%) |
| Mehrmals pro Woche | 1.138 (28,7%) | 44 (29,6%) | 9 (29,7%) |
| Einmal pro Woche | 598 (15,1%) | 8 (5,3%) | 3 (9,2%) |
| Ein- bis dreimal im Monat | 270 (6,8%) | 9 (6,3%) | 1 (4,8%) |
| Seltener | 490 (12,3%) | 16 (10,6%) | 0 (0,0%) |
| Nie | 1.101 (27,8%) | 56 (37,3%) | 4 (13,3%) |
| Derzeitiges Rauchverhalten |  |  |  |
| Ja, täglich | 535 (13,5%) | 12 (7,8%) | 0 (1,0%) |
| Ja, gelegentlich | 174 (4,4%) | 13 (8,5%) | 3 (10,3%) |
| Nein, nicht mehr | 1.501 (37,9%) | 47 (31,1%) | 5 (18,4%) |
| Habe noch nie geraucht | 1.755 (44,3%) | 79 (52,6%) | 20 (70,1%) |
| Häufigkeit von alkoholischen Getränken |  |  |  |
| Täglich | 383 (9,7%) | 6 (4,2%) | 3 (11,6%) |
| Mehrmals in der Woche | 1.069 (27,0%) | 43 (28,8%) | 4 (15,1%) |
| Einmal in der Woche | 597 (15,0%) | 13 (8,7%) | 7 (25,6%) |
| Ein- bis dreimal im Monat | 494 (12,5%) | 19 (12,8%) | 2 (6,1%) |
| Seltener | 981 (24,7%) | 57 (38,1%) | 7 (22,8%) |
| Nie | 442 (11,1%) | 11 (7,4%) | 5 (18,8%) |
| Subjektiver Gesundheitszustand (von 1 = sehr gut bis 5 = sehr schlecht) | 2,4 (0,8) | 2,3 (0,7) | 2,1 (0,9) |
| Anzahl körperlicher Erkrankungen | 2,5 (2,0) | 2,4 (1,5) | 1,8 (1,5) |
| Depressive Symptome (CES-D; 0 bis 45: je höher der Wert, desto mehr depressive Symptome) | 6,2 (5,9) | 5,7 (4,4) | 3,5 (2,7) |

Rundungsfehler bei der Gewichtung sind zu berücksichtigen.

**Tabelle A2**. Migrationshintergrund und Einsamkeit. Ergebnisse basierend auf Welle 7 der DEAS-Studie (adjustierte lineare Regressionen; alle Kovariaten werden dargestellt)

| Variablen | Einsamkeit  (Umgang mit fehlenden Werten: fallweiser Ausschluss) | Einsamkeit  (Umgang mit fehlenden Werten: FIML) |
| --- | --- | --- |
|  |  |  |
| Migrationshintergrund: Migrationshintergrund und persönliche Migrationserfahrung (Referenzkategorie: Kein Migrationshintergrund) | 0,15* | 0,15* |
|  | (0,004 - 0,30) | (0,01 - 0,30) |
| Migrationshintergrund: Migrationshintergrund und persönliche Migrationserfahrung (Referenzkategorie: Kein Migrationshintergrund) | -0,27* | -0,27* |
|  | (-0,52 - -0,02) | (-0,51 - -0,02) |
| Alter (in Jahren) | -0,01** | -0,01** |
|  | (-0,01 - -0,00) | (-0,01 - -0,00) |
| Geschlecht: Frau (Referenzkategorie: Mann) | -0,14*** | -0,14*** |
|  | (-0,20 - -0,07) | (-0,20 - -0,07) |
| Familienstand: - Verheiratet, getrennt lebend (Referenzkategorie: Verheiratet, zusammenlebend) | 0,06 | 0,06 |
|  | (-0,12 - 0,24) | (-0,11 - 0,24) |
| - Geschieden | 0,04 | 0,04 |
|  | (-0,05 - 0,13) | (-0,05 - 0,13) |
| - Verwitwet | 0,04 | 0,05 |
|  | (-0,05 - 0,13) | (-0,04 - 0,14) |
| - Ledig | 0,12* | 0,12* |
|  | (0,02 - 0,22) | (0,03 - 0,22) |
| Bildungsniveau nach ISCED-97: - Mittel (Referenzkategorie: Niedrig) | -0,09 | -0,09 |
|  | (-0,23 - 0,06) | (-0,23 - 0,06) |
| - Hoch | -0,08 | -0,08 |
|  | (-0,22 - 0,07) | (-0,22 - 0,07) |
| Erwerbsstatus: - Im Ruhestand (Referenzkategorie: Aktiv erwerbstätig) | 0,04 | 0,05 |
|  | (-0,05 - 0,14) | (-0,05 - 0,14) |
| - Sonstige nicht Erwerbstätige | -0,08 | -0,07 |
|  | (-0,20 - 0,05) | (-0,19 - 0,05) |
| Haushaltsnettoeinkommen in Euro | -0,00 | -0,00 |
|  | (-0,00 - 0,00) | (-0,00 - 0,00) |
| Sport machen: Mehrmals pro Woche (Referenzkategorie: Täglich) | -0,09 | -0,09 |
|  | (-0,19 - 0,00) | (-0,19 - 0,00) |
| - Einmal pro Woche | -0,06 | -0,06 |
|  | (-0,17 - 0,05) | (-0,17 - 0,05) |
| - Ein- bis dreimal im Monat | 0,06 | 0,06 |
|  | (-0,15 - 0,27) | (-0,15 - 0,27) |
| - Seltener | 0,02 | 0,02 |
|  | (-0,12 - 0,16) | (-0,11 - 0,16) |
| - Nie | -0,05 | -0,05 |
|  | (-0,15 - 0,05) | (-0,15 - 0,05) |
| Derzeitiges Rauchverhalten: Ja, gelegentlich (Referenzkategorie: Ja, täglich) | -0,11 | -0,11 |
|  | (-0,30 - 0,08) | (-0,30 - 0,08) |
| - Nein, nicht mehr | -0,00 | -0,00 |
|  | (-0,12 - 0,11) | (-0,12 - 0,11) |
| - Habe noch nie geraucht | -0,03 | -0,03 |
|  | (-0,14 - 0,08) | (-0,15 - 0,08) |
| Häufigkeit von alkoholischen Getränken: Mehrmals pro Woche (Referenzkategorie: Täglich) | -0,07 | -0,07 |
|  | (-0,19 - 0,05) | (-0,19 - 0,04) |
| - Einmal pro Woche | -0,10 | -0,11 |
|  | (-0,22 - 0,01) | (-0,22 - 0,01) |
| - Ein- bis dreimal im Monat | -0,02 | -0,01 |
|  | (-0,14 - 0,11) | (-0,14 - 0,11) |
| - Seltener | -0,04 | -0,04 |
|  | (-0,15 - 0,07) | (-0,15 - 0,07) |
| - Nie | 0,07 | 0,07 |
|  | (-0,06 - 0,20) | (-0,06 - 0,19) |
| Subjektiver Gesundheitszustand (von 1 = sehr gut bis 5 = sehr schlecht) | 0,06* | 0,07** |
|  | (0,02 - 0,11) | (0,02 - 0,11) |
| Anzahl körperlicher Erkrankungen | 0,03** | 0,03** |
|  | (0,01 - 0,04) | (0,01 - 0,04) |
| Depressive Symptome (CES-D; 0 bis 45: je höher der Wert, desto mehr depressive Symptome) | 0,02*** | 0,02*** |
|  | (0,01 - 0,03) | (0,01 - 0,02) |
| Konstante | 2,27*** | 2,27*** |
|  | (1,88 - 2,65) | (1,89 - 2,65) |
|  |  |  |
| Zahl der Beobachtungen | 4.145 | 4.190 |
| R² | 0,16 | 0,16 |

Unstandardizierte Beta-Koeffizienten werden dargestellt, 95%-Konfidenzintervalle in Klammern, *** p<0.001, ** p<0.01, * p<0.05, + p<0.10; Gewichtung wurde vorgenommen.
